# Hypoxic exosomes alleviated the spinal cord injury after ischemic/reperfusion in a rat model

**DOI:** 10.34172/apb.025.43118

**Published:** 2025-05-30

**Authors:** Mahdiyeh Asadzadeh Bavil, Gollam Hossein Farjah, Bagher Pourheydar, Reza Rahbarghazi

**Affiliations:** ^1^Neurophysiology Research Center, Department of Anatomy, Faculty of Medicine, Urmia University of Medical Sciences, Urmia, Iran; ^2^Stem Cell Research Center, Tabriz University of University of Medical Sciences, Tabriz, Iran; ^3^Department of Applied Cellular Sciences, Faculty of Advanced Medical Sciences, Tabriz University of Medical Sciences, Tabriz, Iran

**Keywords:** Hypoxia, Exosomes, Spinal cord injury, Ischemic/Reperfusion, Regeneration

## Abstract

**Purpose:**

Spinal cord ischemia-reperfusion injury (SCII) is initiated following the occlusion of supporting blood vessels, leading to the loss of neurological function. Here, we aimed to study the regenerative properties of tourniquet-induced hindlimb ischemia exosomes (Exos) in SCII Wistar rats.

**Methods:**

Exos were isolated from rats following tourniquet-induced hindlimb ischemia. CellTracker^TM^ CM-DiI-labeled Exos were injected systematically into SCII rats subjected to 60 min of abdominal aorta occlusion. The distribution of Exos was monitored using an immunofluorescence assay. Using histological examination and real-time PCR analysis, glial cell number, pyknotic and swollen neurons, and expression of apoptosis genes were studied. Oxidative stress was examined by measuring the SOD, GPx activity, MDA, and TAC levels. The neurological assessments were also performed 72 hours after the Exo injection.

**Results:**

Data revealed cup-shaped spherical Exos with average size and zeta potential of 279.3 nm and 15.6 mV, respectively. The isolated particles were CD9+, CD63+, and CD81+, indicating the existence of typical Exo biomarkers. Histological analysis showed reduced gliosis, pyknotic, and swollen neurons compared to SCII rats after Exos injection (*P*<0.05). Data indicated the existence of Exos at the site of injury 24 hours after systemic injection. The injection of hypoxic Exos led to inhibition of apoptosis [Bax (~0.6-fold↓), and Bcl-2 (~3.97-fold↑)] and reduction of oxidative stress [MDA (~58%↓), SOD (~310%↑), GPx (~260%↑), and TAC (~300%↑)] compared to SCII rats (*P*<0.05). Neurological assessments revealed the reduction of withdrawal response and motor deficit index in SCII rats after injection of hypoxic Exos.

**Conclusion:**

Hypoxic Exos are valid regenerative tools for the alleviation of spinal cord injury (SCI) following ischemic/reperfusion.

## Introduction

 Neurological disorders and spinal cord injuries (SCIs) are the most challenging issues in human medicine.^1^ Compulsion, contusion, and incision are the main underlying causative factors of SCI in the clinical setting.^2^ Disability, loss of physical activity, and permanent paralysis, along with socioeconomic burden are the post complications in SCI survivors.^3^ Additionally, spinal cord ischemia is another pathological condition that can occur in individuals with the same post complications such as consistent myelopathy, paraparesis, paraplegia, etc.^4^ Vasculitis, iatrogenic surgical errors, and dissections can increase the possibility of spinal cord ischemia.^5^ Of note, attempts to re-establish blood supplementation and reperfusion by aortic artery surgery can promote the possibility of further neurological damage.^6^ Several strategies such as cerebrospinal fluid drainage, distal aortic reperfusion, and thrombolytic approaches along with other supportive strategies can help to restore, in part but not completely, the function of injured neurons within the ischemic zone.^7^ The re-establishment of blood into the injured ischemic sites can exacerbate oxidative and nitrosative stress, resulting in lipid peroxidation, apoptosis, necrosis, and other cytopathic effects.^8^

 In recent years, regenerative medicine has provided novel therapeutic approaches along with several conventional therapies in the alleviation of neurological injuries.^9^ It has been shown that cell byproducts such as extracellular vesicles (EVs), mainly exosomes (Exos), are valid therapeutic bullets to alter various signaling pathways inside the neuronal cells.^10^ Exos are nano-sized EVs with an average diameter of 30-200 nm produced by the endosomal system and released into the extracellular matrix (ECM).^11^ These particles can easily distribute in several biofluids and are involved in cell-to-cell intercommunication. Due to the existence of several signaling molecules and specific signature profiles, Exos exert their therapeutic effects in the recipient cells.^10,12^

 Exos are actively interchanged between the neurons and glia in the central nervous and peripheral nervous systems, indicating the crucial roles of Exos in neuronal plasticity and functions.^13^ In response to hypoxic conditions, Exos are released into ECM with specific cargo that can trigger distinct signaling pathways in the target sites.^14^ It has been shown that hypoxic Exos differ from normoxic Exos in terms of their cargo, and stimulation/inhibition of specific signaling cascades.^15^ For instance, hypoxia pre-conditioning of mesenchymal stem cells (MSCs) can increase exosomal miR-126 contents with the ability to stimulate the angiogenic, migration, and proliferation capacity of cells in bone injuries.^16^ Mu et al. found that the transplantation of hypoxic Exos with higher levels of hypoxia-inducible factor 1-alpha (HIF-1α) can accelerate the regeneration of SCI via stimulation of vascular endothelial growth factor (VEGF), leading to functional recovery.^17^ In an experiment conducted by Gregorius and coworkers, they found that the injection of hypoxic MSC Exos into the stroke mice led to enhanced microvascular density, neuronal viability, and reduced astrocytic scar formation.^18^ Despite the existence of immense neuroprotective roles, it has not been precisely addressed how and by which mechanisms hypoxic Exos can regulate neuronal recovery after traumatic injuries or ischemic changes.

 Here, in this study, we aimed to explore the neuroprotective properties of hypoxic Exos in a rat model of SCI. To this end, isolated Exos from tourniquet-induced hindlimb ischemia were injected into SCI rats after occlusion of the abdominal aorta into the target sites. We hope that this study can help to develop a *de novo* therapeutic modality for the alleviation of pathological outcomes and stimulation of regeneration inside the injured spinal cord tissue.

## Materials and Methods

###  Ethical issues 

 All procedures of the current study were approved by the local ethical committee of Urmia University of Medical Sciences (grant No: 11148, and the ethical code of IR.UMSU.AEC.1400.003. Animals were treated under the previously published guidelines [Guide for the Care and Use of Laboratory Animals, 8^th^ edition, NIH]. Animals were kept in standard conditions with free access to drinking water and chewing foods. A total of 40 mature male Wistar rats were used in this study. 5 rats were used for the isolation of serum Exos and the rest rats were randomly allocated into five groups as follows; Control, Sham, I/R, I/R + Vehicle, and I/R + Exos groups (each in 7).

###  Exo isolation and enrichment 

 To this end, tourniquet-induced ischemia was done in hind limbs according to the previously published protocols.^19^ Five rats were anesthetized using 100 mg/kg ketamine solution and subjected to 4-5 cycles of 5-minute I/R episodes by tourniquet to restrict the blood supplementation. After that, about 10 ml of blood samples were directly taken from cardiac tissue and allowed clotted. Then, samples were centrifuged at 2000 rpm for 10 minutes to harvest the serum. The collected sera were centrifuged at 300 g for 5 minutes, and 10 000 rpm for 20 minutes at 4 ˚C, and passed through 0.22 µm microfilters to exclude the cell debris and organelles. The procedure was continued with the centrifugation of samples at 100 000 rpm (two times) for 1 hour to eliminate protein contamination and harvest the Exo pellet. The pellets were stored at -80 ˚C until use.

###  Exo characterization and immunophenotyping 

####  SEM and TEM imaging

 To study the ultrastructural features of isolated Exos, SEM and TEM images were taken. In short, Exo samples were placed on aluminum foil and allowed to air-dry. After that, samples were fixed using 2% paraformaldehyde solution and incubated with an ascending concentration of alcohol, 60, 70, 80, 90, 95, and 100, each for 5 minutes. Then, samples were gold-sputtered and monitored using an SEM apparatus (Mira-3 FEG SEM microscope, Tescan Co., Czech). Exos were also characterized using TEM assay. For this end, Exo pellets were resuspended in phosphate-buffered saline (PBS) and placed on carbon-coated 300-mesh copper grids stained with uranyl acetate covered with carbon film, and imaged under 100 kV using (LEO 906, Zeiss, Germany).

###  Western blotting

 To confirm the Exo phenotype, the existence of tetraspanins such as CD9, CD63, and CD81 were monitored using western blotting. For this purpose, Exos were lysed using protein lysis buffer (RIPA), and protein levels were measured by BCA assay. After that, 10 µg of Exo samples were electrophoresed using 10% SDS-PAGE electrophoresis and transferred onto PVDF membrane. Following blocking using 5% bovine serum albumin (BSA), membranes were incubated with anti-CD9 (Cat no: sc-13118), -CD63 (Cat no: sc-5275), and -CD81 (Cat no: sc-166029) for 1 hour at room temperature and washed three times with PBST and incubated with secondary HRP conjugated antibody (Cat no: sc-516102) for 1 hour. Membranes were washed with PBST and immunoreactive bands were visualized using X-ray films and ECL solution.

###  Dynamic light scattering (DLS)

 To measure the hydrodynamic diameter and zeta potential value, DLS was used. In brief, Exo samples were diluted in distilled water and the mentioned parameters were measured using the Malvern Zetasizer Nano ZS system (Malvern; Germany).

###  Exo labelling

 To label isolated Exos, vital fluorochrome staining was done. Exos were incubated with 20 µM CellTracker^TM^ CM-DiI dye (Ref no: C7000; Invitrogen) for 20-30 minutes at 37°C according to previously published data.^11,20^ Freshly isolated Exos were washed twice with PBS, and transplanted into spinal cord ischemic injury (SCII) rats.

###  Immunofluorescence imaging 

 It has been claimed that Exos are mostly cleared from the site of injection a few hours after transplantation.^21^ In this regard, tissues were sampled after 24 hours to confirm Exo distribution at the site of injection. In short, samples were embedded in an optimal cutting temperature medium, and 5 µm-thick cryosections were prepared using the Leica microtome system. After that, the sections were placed on poly-D-lysine-coated slides and subjected to PBS washes. Then, samples were incubated with 1 µg/mL DAPI solution (Sigma-Aldrich) for 30 seconds and washed three times with PBS. The slides were imaged using Olympus microscopy (Model: BX50; Japan).

###  Histological examination

 To monitor histological changes and reparative mechanisms pre- and post-Exo injection into SCII rats, samples were taken after 3 days, fixed in 10% formalin solution.^22,23^ After dehydration, and embedding in paraffin blocks, samples were subjected to hematoxylin-eosin (H & E) staining. To calculate the local density of neurons and glia in spinal cord grey matter, a stereological approach was applied according to the following formula; $N=\frac{\sum Q}{\sum P\times h\times a/f}\times t/BA$ Where ∑Q stands for the number of cells (nucleus); h is the height of the dissector; ∑p is the accumulative number of total cells in the microscopic field. a/f fraction is the area/field ratio. BA is the block advance of microtome and t is associated with the thickness of sections.

###  Real-time PCR analysis

 In this study, the expression of pro-apoptosis (Bax) and anti-apoptosis genes were assessed using real-time PCR analysis. Spinal cord tissues were sampled from different groups and total RNA contents were isolated using a TRIzol. The process was continued with the reverse transcription of isolated RNAs to cDNAs (AddBio; South Korea). The primers targeting Bax and Bcl-2 genes were used based on the previously published data (Table 1). Real-time PCR reactions were performed with 5 µL Master Mix, 0.5 µL Primer Mix (Forward and Reverse), 3.5 µL DEPC-treated Water, 1 µL cDNA from different groups and recommended solutions using the StepOnePlus Real-Time PCR System. The expression of each gene was calculated using 2−ΔΔct, after normalization with GAPDH as a housekeeping gene.

**Table 1:** Primer list

**Target**	**Forward primer**	**Reverse primer**	**Annealing temperature**	**Ref**
*BAX*	GACTCCCCCCGAGAGGTCTT	ACAGGGCCTTGAGCACCAGTT	59 ˚C	^24^
*BCl-2*	GAGCGTCAACAGGGAGATGTC	TGCCGGTTCAGGTACTCAGTC	59 ˚C	^24^
*GAPDH*	GGCAAGTTCAACGGCACAG	CGCCAGTAGACTCCACGAC	59 ˚C	^25^

###  Monitoring oxidative stress 

 The activity of SOD (Cat no: SD125; RANDOX), GPx (Cat no: RS 504; RANDOX), and TAC (Cat no: NX 2332; RANDOX) were measured according to the manufacturer’s instructions. Tissue samples were lysed using protein lysis buffer (RIPA). Exos were incubated with an equal volume of ice-cold RIPA buffer for 30 minutes at 4 °C. Samples were centrifuged at 10 000 rpm and the protein content of supernatants was detected using BCA assay. For the detection of SOD activity, 50 μL of the diluted sample was mixed with 100 µL of Mixed Substrate (R1) followed by the addition of 150 μL Xanthine Oxidase solution (R2). The absorbance of samples was read at 505 nm twice after 30 seconds and 3 minutes using autoanalyzer (Alcyon 300, USA), and the activity of SOD was expressed as Unit/mL/g protein. To detect GPx activity, 20 µL of diluted samples were mixed with 1000 µL working solution, and 40 µL cumene hydroperoxide and the final absorbance was read at 340 nm using an autoanalyzer (Alcyon 300, USA). To measure the MDA levels, the TBARS assay was used according to previously published data.^26^ In brief, 500 µL of homogenized spinal cord sample was dissolved in 3 mL of 1% phosphoric acid solution. After vortexing, 1 mL of 0.675% thiobarbituric acid was added to the test tubes and placed in a boiling water bath for 45 minutes. Then, the test tubes were cooled, 3 mL of normal butanol was added, and vortexed again for 2 minutes. Thereafter, tubes were centrifuged for 10 minutes at 3000 rpm. After separating the organic phase (supernatant), optical density was measured at a wavelength of 532 nm. By comparing data to a standard curve, the serum MDA levels were calculated. TAC was also assessed in samples by the mixing of 20 µL diluted samples with chromogen buffer containing metmyoglobin, and ABTS, and the final absorbance was read at 600 nm. The levels of MDA, TAC, and activity of SOD and GPx were expressed per gram of protein.

###  Exo injection in SCII rats and neurological assessments 

 For this purpose, rats were restrained and hypoxic Exo samples (~130 µg/mL) were directly injected into a caudal vein before deep anesthesia and allowed rats to rest for 20 minutes at standard conditions.^11^ After that, rats were anesthetized as mentioned above. The rat model of SCII was induced by using an aortic cross-clamping method as previously described.^27^ Rats were fixed in the supine position, and the surgical incision was made in the ventricle midline to expose the aorta after laparotomy. The distal aorta was clumped using bulldog forceps after renal arteries for 60 minutes. Thereafter, the clumps were removed and ensure the reestablishment of the blood supply. Finally, the surgical incisions were closed using 4-0 sterile suture nylon threads. At distinct time points, 24, 48, and 72 post-surgery several neurological parameters such as motor deficit indices (ambulation, placing/stepping reflex) and sensory deficits index (withdrawal reflex latency) were assessed.

###  Statistical analysis 

 Data are expressed as mean ± SD. The statistical differences were assessed using one-way ANOVA with Tukey post hoc analysis. *P* < 0.05 was considered statistically significant.

## Results

###  Exo characterization and immunophenotyping

**Figure 1:**
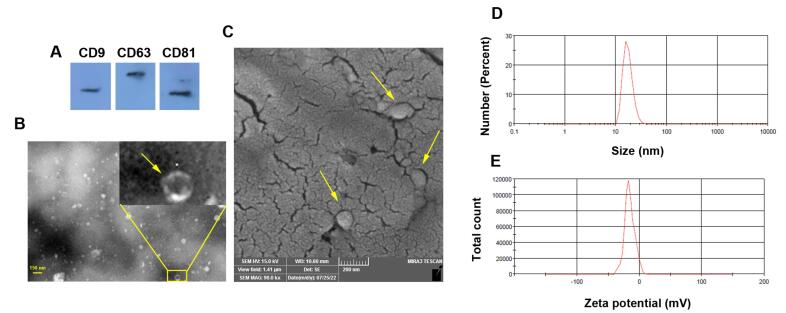
Characterization of rat Exos using western blotting (A; CD9, CD63, and CD81), TEM (B; yellow arrows), SEM (C; yellow arrows), and DLS analysis (D-E)

 To confirm the Exo phenotype, several assays were used (Figure 1A-E). Western blotting indicated the existence of tetraspanins (CD9, 63, and 81) in enriched exosomal pellets obtained by ultracentrifugation (Figure 1A). TEM images indicated nano-sized cup-shaped Exos with heterogeneous populations and sizes (Figure 1B). Likewise, the SEM technique exhibited a spherical shape Exo with different diameter sizes embedded in a gold-sputtered background (Figure 1C). The hydrodynamic diameters and zeta potential values of isolated Exos were measured using DLS techniques, according to our data, Exo exhibited mean size and zeta potential of 279.3 nm and 15.6 mV, respectively (Figure 1D-E). These features indicate the typical Exo phenotype with relevant physicochemical properties.

###  Labeled Exo were successfully tracked in ischemic spinal cord tissue 

**Figure 2:**
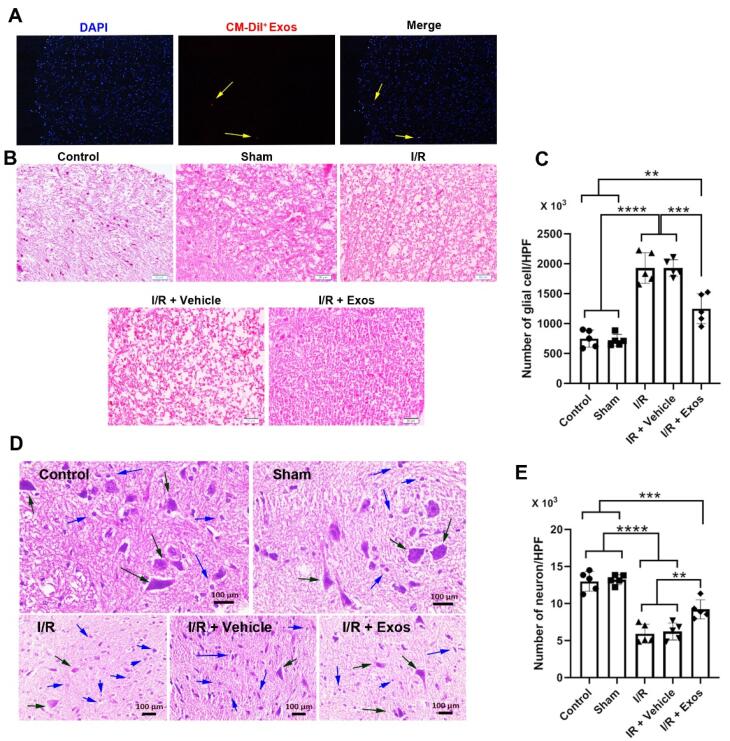
Immunofluorescence analysis of red-colored CellTracker^TM^ CM-DiI + Exos in spinal cord tissue 24 hours after being transplanted (A; yellow arrows). Histological analysis of spinal cord tissue in different experimental groups using H & E staining (B). Calculation of glial cells in the spinal cord of different groups (C). Monitoring the number of pyknotic neurons (blue arrows) and swollen neurons (black arrows) in the spinal cord section stained with H & E solution (panels D and E). One-way ANOVA with Tukey post hoc analysis. **P < 0.01; ***P < 0.001; ****P < 0.0001

 To track the injected Exos in the injured spinal cord tissue, we used fluorescence CellTracker^TM^ CM-DiI dye. IF images indicated the existence of red-colored Cell Tracker^TM^ CM-DiI + Exos within the injured spinal cord tissue (5 µm-sized section) after 24 hours indicated by numerous blue-colored DAPI + nuclei (Figure 2A). These data indicated the distribution of injected Exos with the neuronal tissue.

###  Exos reduced aberrant histological changes in SCII rats

 H & E staining was done to monitor histological changes before and after I/R and Exo injection (Figure 2B-D). Bright-field images indicated the existence of a swollen appearance in I/R, I/R + Vehicle groups compared to sham and control samples (Figure 2B). Of note, the injection of Exos reduced the swelling rate in SCII rats and closed it to the control levels. These data exhibit that Exos can control aberrant histological changes indicated by the regulation of swelling in the white matter of spinal cord tissue. The induction of hypoxic conditions led to an increase in glial cell number compared to the Control and Sham rats Figure 2C; *P* < 0.0001). Data also showed the reduction of glial cells at the site of ischemia in rats that received Exos compared to I/R and I/R + Vehicle groups (Figure 2C; *P* < 0.001). Despite the reduction of glial cells in Exo-treated rats, the number of glial cells was significantly high as compared to the Control and Sham rats (*P* < 0.01). These data indicated the anti-inflammatory properties of Exos in rats with SCII over time. Data also showed pathological changes in neuronal cells with pyknotic changes in neurons within the spinal cord parenchyma (Figure 2D; black arrows). The ischemic neurons are shown in the bright-field images with the lack of a nucleus compartment and loss of normal morphologies in which most neurons in the ischemic groups are swollen and round-shaped. Along with these changes, round-shaped microglia can be detected in the proximity of injured neurons. The injection of Exos can in part but not completely restore the neuronal cell morphologies in which a small number of injured neurons are evident in images (Figure 2D). Besides, the number of local microglia was also reduced in Exo-treated SCII rats. Statistical analysis proved the therapeutic effects of Exos in the restoration of neuron density in spinal cord tissue exposed to ischemia. In hypoxic spinal cord tissue, the number of neurons was significantly reduced compared to the Control and Sham rats (*P* < 0.0001; Figure 2E). Exos can increase the number of neurons per high-power field as compared to the I/R, and I/R + Vehicle groups (*P* < 0.01).

###  Exos reduced apoptotic changes and oxidative stress in SCII rats 

**Figure 3:**
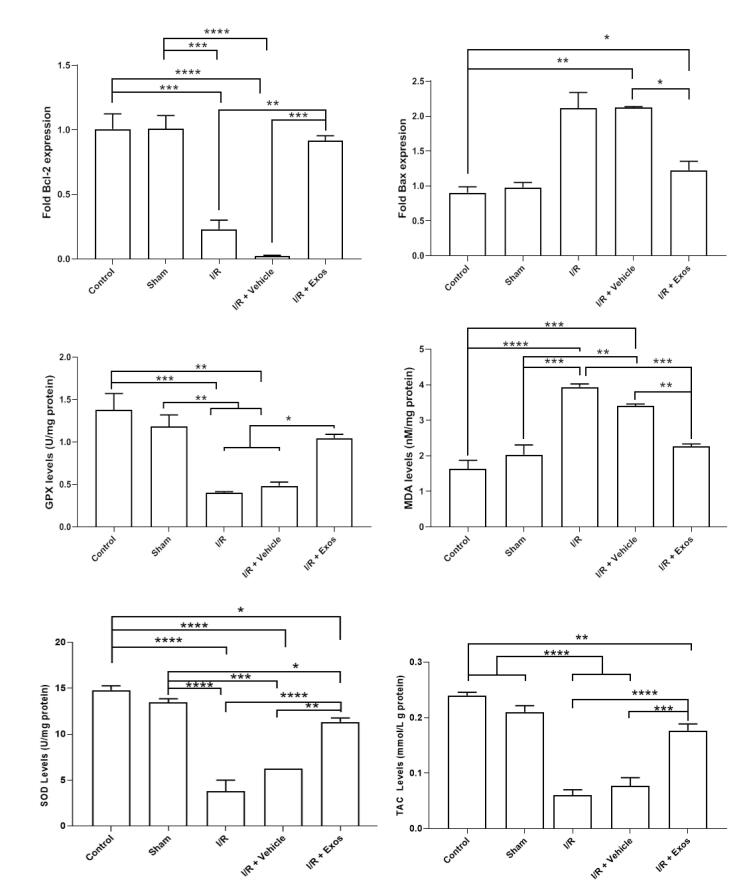
Measuring the expression of pro-apoptosis (Bax) and anti-apoptosis (Bcl-2) genes in spinal cord samples using real-time PCR assay (n = 3). Monitoring oxidative stress status (GPx, MDA, SOD, and TAC) in SCII samples after injection of Exos. One-way ANOVA with Tukey post hoc analysis. *P < 0.05; **P < 0.01; ***P < 0.001; and ****P < 0.0001

 To assess the level of apoptotic changes and oxidative stress, the expression of Bax, and Bcl-2, and the activity of enzymes such as SOD, GPx, TAC, and MDA content. Real-time PCR analysis indicated that the induction of SCII the apoptotic changes by the down-regulation of BCl-2, and up-regulation of Bax in the I/R and I/R + Vehicle rats compared to the control group (*P* < 0.05; Figure 3). The injection of Exos into the target sites reversed these effects and was close to the control levels in which non-significant differences were obtained compared to the control rats (*P* > 0.05). Monitoring the oxidative stress status indicated the activity of SOD, and GPx were significantly reduced in I/R, and I/R + Vehicle rats compared to the control rats (*P* < 0.05) while the injection of Exos led to the increase of these enzymes in SCII rats. Despite these changes, statistically significant changes were obtained in these groups compared to the control group (*P* < 0.05). Along with these changes, the elevation of MDA levels coincided with the reduction of TAC, indicating the active oxidative status. In the I/R + Exos group, the levels of MDA were reduced and TAC capacity increased, indicating the restoration of antioxidative mechanisms. These features indicate that Exos can alleviate the SCII in rats via the modulation of oxidative stress.

###  Exos improved neurological performance in SCII rats 

**Figure 4:**
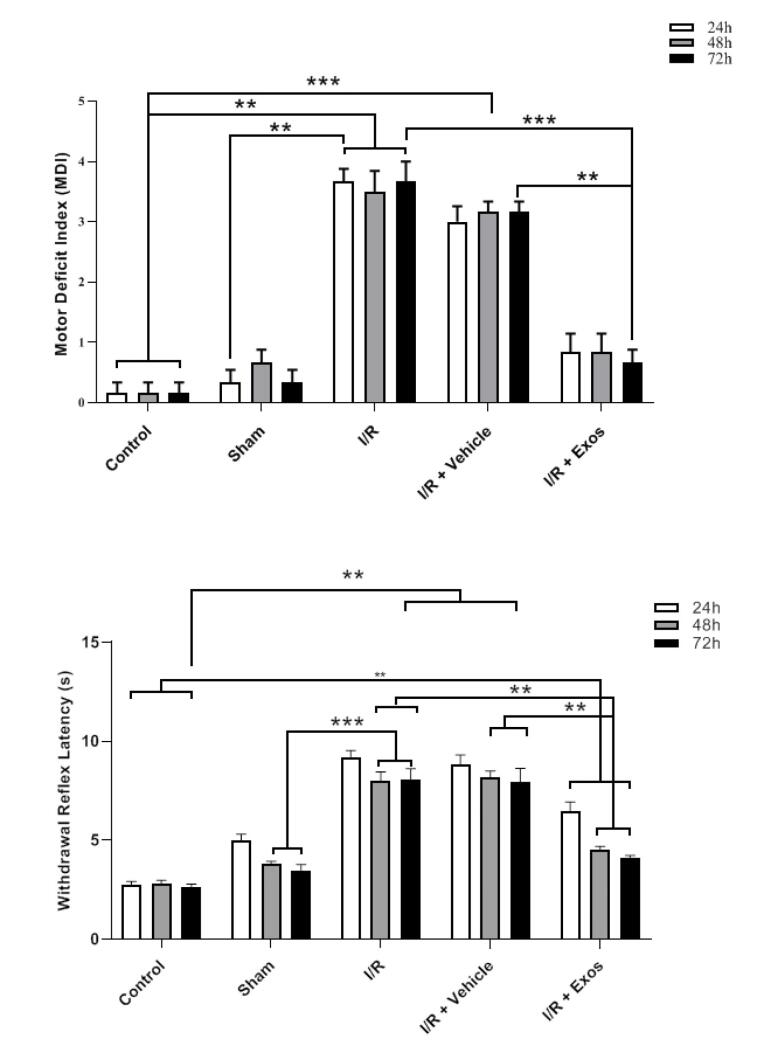
Neurological performance (MDI and withdrawal reflex) in SCII rats 24, 48, and 72 hours after the injection of Exos. One-way ANOVA with Tukey post hoc analysis. **P < 0.01; and ***P < 0.001

 Data indicated that MDI values were increased in rats after being to ischemic changes in the spinal cord tissue during the 72 hours compared to the control (Figure 4). A similar trend was achieved in SCII rats that received the Vehicle. According to the obtained data, the injection of Exos in SCII rats contributed to the reduction of the MDI index and was close to the control levels in which no statistically significant differences were obtained related to the control rats (*P* > 0.05). Along with these changes the enhanced WRL indices in SCII rats, either I/R or I/R + Vehicle group, were reduced after the injection of Exos (*P* < 0.05). These data indicate that Exos are eligible therapeutics to alleviate the SCII in rats and return to near-to-control levels.

## Discussion

 SCII is a devasting pathological condition in the clinical setting and is commonly induced due to occlusion of the thoracic or thoracoabdominal aorta.^28^ These features can contribute to the loss of neurological function.^29^ Despite recent progress in using novel therapeutic modalities, the pathological conditions associated with SCII could not be completely removed. Thus, it is recommended to develop new therapeutic approaches for the alleviation of injured spinal cord tissue and restoration of neurological function. Here, the regenerative potential of hypoxic Exos were assessed in a rat model of SCII induced by using an aortic cross-clamping method. Data indicated the existence of redo-colored Dil-labeled Exos at the site of injury after 24 hours. However, we did not assess how much fraction of injected Exos reached the target sites. Currently, various techniques have been used for labeling Exos for in vitro and in vivo studies.^30^ In different studies, exogenous labeling using fluorochromes and luciferins, radioactive tracers, and contrast agents are common while endogenous labeling of Exos in the parent cells using Cre-lox and CRISPR-Cas systems are powerful techniques for tracking Exos.^30^ Application of lipophilic tracers such as Dil provides an inexpensive, fast, friendly-use method to monitor the kinetics of transplanted Exos in the biological system.^31^ However, partial dye leakage is possible and this phenomenon is intensified when the integrity of Exos is lost.^32^

 We also showed that the number of glial cells, pyknotic, and swollen neurons was reduced in SCII rats about 72 hours after Exo injection. It has been shown that hypoxia can produce Exos with anti-inflammatory and angiogenesis properties.^33^ The reduction of glial cells would be that Exos harbor several signaling molecules with the potential to influence cell apoptosis, pyroptosis, and inflammation under ischemic conditions.^34^ In an experiment, the preconditioning of MSCs produced Exos with certain cargoes such as miR-216a-5p that resolve inflammation in traumatic SCI by promoting microglial polarization from M1 to M2.^35^ Several studies have confirmed the protective effects of limb remote ischemic preconditioning on SCII.^36,37^ In response to hypoxic conditions, it is also possible astrocytes acquire the A2 phenotype in the presence of Exos via the stimulation of autophagic response.^38^ The production and release of several cytokines by A2 astrocytes can exert neuroprotective effects by the stimulation of synaptogenesis, neuron survival, and growth.^39^ It seems that the production of Exos from a heterogeneous cell population located in the hind limb can be distributed toward the ischemic site in response to several cytokine gradients.^40^ The present data also showed the reduction of pyknotic and swollen neurons at the site of SCII after injection of hypoxic Exos. Along with these findings, the expression of Bcl-2 and Bax was significantly up-regulated and down-regulated respectively compared to I/R + Vehicle rats. Based on the previous data, EVs possess the potential to reduce the number of dead neurons coinciding with the MAP2 + cells in the penumbra.^41^ These features contribute to the reduction of apoptotic changes (Bax↓, and Bcl-2↑) as indicated by real-time PCR analysis. The reduction of oxidative stress (SOD, GPx, MDA) and increase of TAC in I/R + Exos was also evident. In an experiment conducted by Wang et al., they found that hypoxic umbilical cord MSC Exos can reduce the apoptosis changes in SCII rats via the modulation of circOXNAD1/ miR-29a-3p/ FOXO3a axis.^42^ Based on the data, the levels of pro-inflammatory cytokines such as IL-1β, IL-6, and TNF-α were decreased, leading to hindlimb motor function.^42^ Several experiments have shown that Exos can harbor several anti-oxidant effectors such as SOD1, GPX1, GSH, thioredoxin reductase 1, etc. The levels of these enzymes and genetic materials differ based on the parent cells. Exos can effectively blunt oxidative stress by neutralization free reactive oxygen radicals and preventing the propagation of tissue injury.^43^ Thus, the reduction of SOD, GPx, and MDA in I/R + Exos rats can be related to blunting effects of injected Exos and the reduction of free radicals by glial cells.^44^ The reduction of apoptotic changes, oxidative stress, and gliosis were the most prominent phenomena assessed in this study. These features led to the improvement of the healing process and neurological performance. The current study faces several limitations and shortcomings that need to be precisely elucidated by future experiments. In this study, the isolation of Exos was performed using ultracentrifugation method. This method can yield Exos with some morphological deformities.^45^ Besides, this method co-isolates different subsets of EVs which can overlap the function of Exos after being transplanted into the target sites.^46^ The lack of standard guidelines for the application of Exos with certain doses can lead to inconsistency in obtaining data from different experiments.^47^ It is highly recommended to examine the regenerative potential of Exos from other sources, i.e. stem cells, in the alleviation of SCII models. Using accurate analytical methods, deep-proofing of hypoxic Exo cargoes in comparison with normoxic Exo counterparts can help us find the underlying mechanisms and insights into the function of Exos in SCII cases. Monitoring SCII rats over a prolonged period is mandatory to justify the lack of any side effects and functional recovery after transplantation of Exos. More advanced labeling techniques with higher sensitivity and specificity are suggested to monitor the dynamic of Exos in the biological system. Even though, these approaches enable us to approximately determined which fraction of Exos are internalized by neurons rather than glia. Future studies should also address precise retention time and Exo distribution pattern in SCII model.

## Conclusion

 In this study, it was shown that Exos are a valid alternative to cell-based therapies in central nervous system injuries. Data indicate the reduction of I/R-related injuries in SCII rats via the reduction of oxidative stress and neuronal cell apoptosis. The transplantation of Exos can circumvent several problems and drawbacks associated with direct injection of allogeneic cells either stem cells or mature cell types for the regeneration of SCI injuries.^47^ To translate the numerous preclinical findings, it is mandatory to produce clinical-grade Exos for SCII patients.

## Competing Interests

 Authors declared that there is no conflict of interest related to this study.

## Ethical Approval

 All processes of this study were approved by the local ethics committee of Urmia University of Medical Sciences (IR.UMSU.AEC.1400.003).
